# Characteristics of Dietary Supplements with Folic Acid Available on the Polish Market

**DOI:** 10.3390/nu14173500

**Published:** 2022-08-25

**Authors:** Marta Czarnowska-Kujawska, Joanna Klepacka, Olga Zielińska, María de Lourdes Samaniego-Vaesken

**Affiliations:** 1 Department of Commodity and Food Analysis, The Faculty of Food Sciences, University of Warmia and Mazury in Olsztyn, 10-726 Olsztyn, Poland; 2 USP-CEU Group of Excellence “Nutrition for Life”, ref: E02/0720, Department of Health and Pharmaceutical Sciences, Faculty of Pharmacy, Universidad San Pablo-CEU, CEU Universities, Montepríncipe Urbanization, 28660 Boadilla del Monte, Spain

**Keywords:** folic acid, dietary supplements, HPLC, food authenticity, neural tube defects, pregnancy

## Abstract

One way of increasing folate status, especially in a state of increased demand (e.g., women of childbearing age), is dietary supplementation with folic acid (FA). The dietary supplements market in Poland shows a controversial situation and, for many reasons (the ease of placing them on the market, the lack of control of chemical composition), the possibility of inaccurate information provided on the supplement’s label arises. We questioned whether FA supplements available in Poland are indeed complying with regulations and if they could actually improve folate status amongst the target population groups consuming them. Almost 500 products containing FA were identified and available for sale in pharmacies, all of them including specific information provided by manufacturers on the packaging, such as the amount of FA, their intended use and daily dosage. HPLC analysis of FA content in 30 randomly purchased supplements exposed that in four of the tested products, FA content was less than 4% of the declared value (DV). Another 11 samples exposed that the difference with declared FA content varied from 25% up to 80% of the DV. The obtained results are in agreement with the ones from inspections previously conducted on the Polish dietary supplements market and indicate the urgent need to implement improvements in the notification system as well as the monitorization of these product’s authenticity.

## 1. Introduction

The present work was inspired by the results of the Report on the Polish dietary supplements market presented by the Polish Supreme Audit Office in 2017. An inspection conducted between 2014 and 2016 showed that the safety level of dietary supplements was not sufficiently provided [1,2]. Directive 2002/46/EC of the European Parliament and of the Council of 10 June 2002 [3] defines “food supplements” as “foodstuffs the purpose of which is to supplement the normal diet and which are concentrated sources of nutrients or other substances with a nutritional or physiological effect, alone or in combination, marketed in dose form, namely forms such as capsules, pastilles, tablets, pills and other similar forms, sachets of powder, ampoules of liquids, drop dispensing bottles, and other similar forms of liquids and powders designed to be taken in measured small unit quantities”. It emphasizes that in order to ensure a high level of protection for consumers, the products that will be placed on the market must be safe and bear adequate and appropriate labeling [3]. Meanwhile, in the conclusions from the Report by Polish authorities [1], the food supplements market was identified as a key risk area, inadequately assessed and supervised by the State institutions responsible for food safety. It was found that not only was the supervision over the health quality of dietary supplements ineffective, but the nutritional education concerning these products was also insufficiently implemented. This is mainly due to the deficient legislative solutions enforcing the introduction and advertising of supplements to the market [1,2]. Currently, a supplement can be placed on the market only after declaring its composition to the health authorities, the Chief Sanitary Inspectorate (GIS) in Poland, through a so-called “notification” [4]. According to the Supreme Audit Office, theoretically, there are chances that the product that is initially placed on the market will be tested, but in practice, the scale of the market exceeds the current control capabilities of the Sanitary Inspections. Controls carried out by the Inspection concern only a part of the market and the control procedures might even take up to several years to complete. In many cases, commercialized dietary supplements are not tested at all [1,2]. It seems that the dynamic expansion of the dietary supplements market does not go hand in hand with the development of effective tools for their control.

The inspection of the Supreme Audit Office showed that amongst dietary supplement sales, including online and also in pharmacies in addition to reliable preparations, there were also adulterated food supplements, containing, for example, prohibited substances from the psychoactive list, or stimulants structurally similar to amphetamines, which can act like drugs [1]. Moreover, testing the samples of supplements to compare their actual content with the composition declared on the product label showed an inappropriate quality, as the samples in question did not display the properties declared by the manufacturers [1]. Consequently, can consumers trust that the supplements they buy actually contain bioactive substances, such as vitamins, minerals, proteins, phytochemicals, antioxidants, etc., in the amounts declared on the product packaging?

Dietary supplements containing folic acid (FA) are a relevant case of study due to their popularity amongst women of reproductive or childbearing age: those who are either planning a pregnancy or those who are already pregnant. In 1991, the randomized trial performed by the Medical Research Council (MRC) Vitamin Study Research Group [5] demonstrated that FA supplementation starting before pregnancy could prevent neural tube defects (NTDs) in the fetus. Anencephaly and spina bifida are two of the most common and serious congenital malformations recognized as NTDs; these severe conditions are incapacitant to the newborn and even incompatible with life [6]. Unfortunately, NTDs affect approximately one in a thousand pregnancies in Europe [7] and at present, most FA supplements available to women of childbearing age are formulated to contain the recommended daily allowance (RDA) of 400 µg per dose [8]. The aim of the current study was to evaluate the assortment of folic acid dietary supplements available on the Polish market and the assessment of their FA content in selected dietary supplements by using a validated HPLC technique.

## 2. Materials and Methods

### 2.1. Folic Acid Supplements Database

The analysis of the Polish market of dietary supplements containing FA was conducted based on the assortment available on the websites of two of the largest pharmacy retailers [9,10]. Data were collected throughout May 2022, and it was based on the information provided by the manufacturers of supplements on pharmacy’s websites. Only those dietary supplements available for sale and with complete information about the product were included in our study. Products were characterized on the basis of their composition, the amount of FA declared per dose (declared value (DV)), the form of occurrence, intended use, price per package and unit. Dose was defined as one tablet, effervescent tablet, capsule, or jelly; and in the case of powdered supplements, one sachet, the weight of which is indicated on the packaging by the manufacturer, and in the case of liquid supplements, the portion per milliliters (mL). In summary, the compiled information included only those dietary supplements that were available for purchase with comprehensive information about the product.

### 2.2. Materials and Reagents

Test materials comprised a total of thirty dietary supplements containing FA, of which eight were single-ingredient supplements containing only FA, twelve were multi-ingredient supplements containing other vitamins and bioactive ingredients, and ten were multi-ingredient liquid supplements. All tested products were chosen based on their positioning on the website of one of the largest pharmacy retailers in Poland. After weighing, supplements in the form of tablets were crushed and thoroughly ground in a mortar, those in capsule form were dissolved in an extraction buffer while stirring on a magnetic stirrer, and liquid supplements were added directly to centrifugal flasks after mixing.

Folic acid standard was obtained from Sigma Aldrich (St. Louis, MO, USA) and prepared as described by Konings [11]. Acetonitrile used for analysis was of HPLC grade, while other chemicals were of analytical grade. Water was purified in the Mili-Q system (Millipore; Vienna, Austria).

### 2.3. Sample Preparation and Folic Acid Quantification

Samples were analyzed in triplicate. Supplements which were in the form of tablets and capsules were weighed in an amount corresponding to the weight of a single tablet or capsule; samples in liquid form in the amount of 10 mL were poured into 30 mL centrifuge flasks (30-mL PPCO Oak Ridge PPCO Nalgene centrifuge tube; Rochester, NY, USA). Then, 20 mL of an extraction buffer (0.1 M phosphate buffer, pH 7.0, with 1% (*w/v*) sodium ascorbate and 0.1% (*v/v*) 2-mercaptoethanol) were added and shaken (2500 rpm/10 s Vortex 4 basic IKA Vortex 4 basic; Staufen, Germany). After boiling in a water bath for 15 min and cooling in ice, samples were centrifuged twice at 12,000 rpm/4 °C/20 min (MPW-350R; Warsaw, Poland). Each time, supernatants were collected in 50-mL amber volumetric flasks filled up with the extraction buffer. The extract was filtered through filter paper, flushed with nitrogen, and then stored until HPLC analysis at −70 °C.

Sample purification using solid phase extraction (SPE) and HPLC analysis were carried out as previously described [12]. In short, the chromatographic separation of FA was carried using the Shimadzu Nexera-i LC-2040 C plus HPLC system (Shimadzu Co.; Kyoto, Japan) and the C18 LC column (150 × 4.6 mm, 3 µm, Luna 100 Å; Phenomenex; Torrance, CA, USA). Briefly, conditions for binary gradient elution, with 30 mM phosphoric acid buffer (pH 2.3) and acetonitrile used as the mobile phases were as follows: starting with 6% (*v/v*) acetonitrile maintained isocratically for the first 5 min, then raised linearly to 25% within 20 min. Separation time—42 min; the flow rate—0.4 mL/min; injection volume—20 µL; column temperature—25 °C. Peaks in the samples were identified based on standard retention time. Quantification of FA was based on UV detection (290 nm) using the external multilevel (*n* = 8) calibration curve with the linear range of 2 ng/mL–9 µg/mL (correlation coefficient >0.9996). The variation obtained between the replicates for each analyzed sample was lower than 10%. Recovery tests were performed by adding known amounts of FA before the extraction with the mean recovery (*n* = 4) obtained at the level of 92% ± 7. The repeatability of the analytical procedure was checked on different extraction days.

## 3. Results and Discussion

### 3.1. Market Analysis of Folic Acid Supplements

Folate, also known generically as vitamin B9, is a water-soluble vitamin which naturally occurs in many foods, such as green leafy vegetables, eggs, liver, legumes, grains and nuts [13,14,15]. Folic acid (FA) is the synthetic form of folate used for food fortification and in dietary supplements; other synthetic forms include L-methylfolate calcium and (6S)-5-methyltetrahydrofolate glucosamine [16]. The generic term ‘folate’ includes both natural folates from food sources and the synthetic form, FA. Folates cannot be synthesized by the body and must, therefore, be provided in the diet [13,14,15]. The daily recommendations for folate intake depend on age, gender and physiological condition and are higher for pregnant and lactating women (Table 1). According to the Polish Society of Gynecologists and Obstetricians Guidelines, women of childbearing age planning a pregnancy should additionally use folate supplementation for a period of at least twelve weeks prior to conception and continue throughout pregnancy, the postpartum period and breastfeeding [17]. It is estimated that the effective absorption in the digestive tract of dietary folates does not exceed 50%. Meanwhile, the absorption of synthetic FA from a dietary supplement can reach up to 100%. Considering the differences of folate bioavailability from different sources, the dietary folate equivalents (DFE) equivalence was established, where 1 µg DFE is equal to 1 µg of dietary folates = 0.6 µg of FA from fortified foods or dietary supplements consumed with foods = 0.5 µg of FA from dietary supplements taken on an empty stomach [18].

In the current situation of low folate status observed in Europe, dietary supplementation with FA is commonly recommended, especially in those states of increased demand [19]. FA is crucial in DNA replication and repair, methylation and synthesis of nucleotides, as well as in the metabolism of other vitamins and amino acids [20]. Low folate status in all population groups has been linked to a number of health problems, such as megaloblastic anemia, increased cardiovascular risk and colorectal cancer, as well as neurocognitive decline in the elderly [21,22,23,24,25]. Maternal folate deficiency is associated, as already mentioned, with a higher risk of NTDs, in addition to cleft lip and palate and Down’s syndrome [26]. Moreover, other complications of pregnancy such as miscarriages, inhibition of intrauterine growth and pre-eclampsia may occur more frequently [27,28]. Data analysis indicates that in Poland, daily folate intakes in adults is at an average level of 110–352 μg per day, while among young non-pregnant women it is approximately 127–315 µg per day. Folate intakes among those aged sixty and above was 133–284 µg per day [29]. Moreover, results from a national survey conducted in the UK between 2008 and 2017 showed that around 90% of childbearing aged women had a folate status below the level recommended to reduce the risk of NTDs. In addition, an estimated 28% of girls and 15% of boys aged 11 to 18 years and 7% of adults had low blood folate levels, which increases their risk of anemia [30].

**Figure 1:**
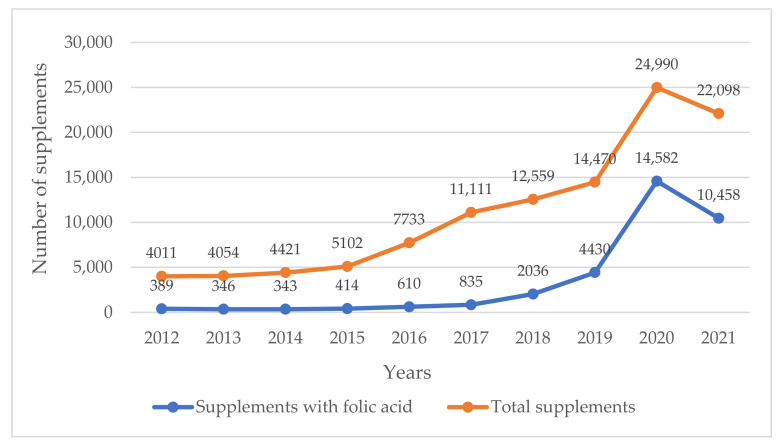
Number of supplements with the notification of the first appearance on the market in the years 2012–2021 based on Chief Sanitary Inspectorate data [30].

Meanwhile, in Poland, the market of dietary supplements, including those containing FA, is developing very dynamically, which is undoubtedly influenced by their widespread advertising [1,31]. It is estimated that, currently, over 65% of Poles use dietary supplements, most often without consulting a doctor or pharmacist. The most popular are vitamin and mineral preparations. There are already nearly 30,000 entities producing and selling dietary supplements, and the market value is estimated at EUR 1.25 billion [32]. According to the Act of 25 August 2006 on food and nutrition safety, dietary supplements and fortified foods are subjected to the obligation to notify the Chief Sanitary Inspectorate of their initial placement on the market [4]. According to a register run by the Sanitary Inspectorate, in 2020, the highest number of more than 14,500 notifications of supplements with declared addition of FA was recorded, and it was more than 58% of all notified products that year (Figure 1) [31]. These numbers, however, indicate the products notified, but it remains unknown how many of them were actually placed on the market, how many and after how long were they withdrawn from sale by the manufacturer.

Consumer opinion polls conducted in Poland show that pharmacies are the most frequent place for purchasing supplements, and account for 65% of sales [32,33]. Based on the analysis of assortment available in the two largest pharmacy retailers in Poland (May 2022), 470 dietary supplements with FA were available for sale [9,10]. Most of these were multi-ingredients products containing, often, other vitamins and minerals (94.5%). Nonetheless, only 26 supplements (5.5%), were mono-ingredient and contained only FA. The most popular form of supplements with FA addition were tablets (36.4%) and capsules (35.7%). Less than 10% of evaluated products were in the form of effervescent tablets, in liquid and powder. Other formats, such as lozenges, jellybeans, lollipops and soluble gums, constituted only 6.4%. Most of the analyzed products were intended for adults to supplement their daily diet with vitamins and minerals. The second most numerous group were products intended for pregnant women, breastfeeding women or those planning pregnancy (Table 2). The vast majority of available supplements (85.7%) contained FA in the form of pteroylglutamic acid (PGA). However, as regulated by the Polish Minister of Health in the area of the composition and labeling of dietary supplements [34], FA can be also added in the form of L-methylfolate calcium and (6S)-5-methyltetrahydrofolate glucosamine salt. The main differences between these chemical forms used as salts in supplement formulation are related to their bioavailability, or proportion of the ingested folate that is accessible and absorbed in the intestinal lumen and is therefore available for metabolic processes or storage [35]. Their metabolic use and fate in the one-carbon (monocarbon) metabolism where they exert their essential functions is related to nucleotide synthesis and red blood cell formation, amongst others [13,35]. PGA was considered to have the highest bioavailability, but currently research has shown that also L-methylfolate calcium and (6S)-5-methyltetrahydrofolate glucosamine can have a similar absorption rate [16]. In the analyzed assortment, 67 supplements contained the methyl folate form alone or in combined addition with PGA. The use of this form of folates, naturally occurring in the blood plasma, results from the controversy related to the potential adverse effects of excessive consumption of synthetic FA, such as masking low status of vitamin B12. A further possible risk is the unknown long-term effect of synthetic PGA not metabolized in the body [36,37]. The amount of FA in analyzed supplements ranged from 12 µg to as high as 2 mg in one dose. The most numerous were supplements with the declared FA content (DV) of 200 µg per dose (29.8%) and 400 µg per dose (26.6%). In twelve products (2.5%), FA was in the amount of 600 µg per dose; in seventeen supplements (3.6%)—800 µg per dose. One analyzed supplement had a declared FA content of 1 mg per dose, which is the considered maximum safe intake of FA from supplements per day [38], and one contained as much as 2 mg of FA. Moreover, according to the Resolution No. 7/2019 [39], the Polish Chief Sanitary Inspectorate’s Dietary Supplements Panel, which is a consultative and advisory body of the Chief Sanitary Inspector in Poland, set the maximum amount of FA in the recommended daily dose at 600 µg for adults and 800 µg in supplements designed for pregnant women. For dietary supplements containing ≥800 µg of FA, it is recommended that the warning: “pregnant women should consult a doctor before use” should appear on the label. Within the analyzed assortment, only three products followed the recommendation, not including those supplements with the highest declared folate content of 1 mg and 2 mg. As a comparison, in the FA dietary market analysis from 2012 [40], 326 supplements with FA were found and similarly to our results; most of them were in the form of multi-ingredients products, in the form of tablets and capsules. More than half of the supplements contained from 200 to 400 µg FA in one dose. Similarly at that time, the most numerous group were products intended for use by adults in order to supplement their daily diet and intended for women planning to be or already pregnant. Additionally, the presence of L-methylfolate calcium was declared only in two supplements, while the others contained PGA [40].

### 3.2. Folic Acid Content Analysis

As previously mentioned, such a large offer of easily available dietary supplements, including those with FA, raises the question of their quality and authenticity. Table 3 presents the results of the analysis of FA content in thirty purchased supplements determined using an HPLC technique. In all thirty tested samples, except for one, the differences in the content of FA were related to a lower content than the manufacturer’s declared value (DV). The first category were eight supplements containing only FA with a dose of 400 µg. All supplements in this category were intended, as clearly stated on the product packaging, for women planning pregnancy or already pregnant. One product had the information that was also intended for smokers, elderly people, women who use oral contraceptives and people who abuse alcohol—all groups characterized as being deficient in FA. In three out of eight one-ingredient supplements, the difference between the determined and the declared vitamin content did not exceed 10%. Conversely, in another different five, the difference ranged from 20% to almost 28%.

Multi-ingredient supplements in the form of tablets, capsules or liquids contained mainly other vitamins apart from FA, even over twenty, and over a dozen different minerals with an exact specification of their amount in one dose on the label [9,10]. In the category of multi supplements in tablets and capsules, declared FA content ranged from 200 to 600 μg per dose. In two out of twelve supplements, determined FA content contained no more than 4 μg despite declaring 400 μg (Table 3). Unfortunately, these were vitamin complexes intended for women planning pregnancy. In the next four products, differences with the declared FA content were at the level of 35–68%, and in another four, from 16% to 21%. Only one supplement (No. 19 in this category) had a vitamin content similar to that specified on the packaging by the manufacturer. In the last category of multi-ingredient supplements in liquid, declared FA content was given in the recommended portion (in mL) and ranged from 30 to 290 μg per dose. The tested products in this category were intended primarily for seniors and adults who want to “improve their vitality, strength and energy”. In this group, two supplements out of ten, No. 21 and No. 28, had similar FA content to that declared. Unfortunately, in two supplements the difference between content determined and specified on the label reached 97.5% for No. 27, and 99.3% for No. 29. In the next four supplements in this group, it was determined that over half of products showed less FA than declared. 

Based on the conducted market analysis, there are nearly 500 FA-containing supplements available in pharmacies, a large number of which theoretically would cover the daily requirements of this vitamin. More than 30 years have passed since the publication of the study that revolutionized modern day supplementation strategies, confirming that the majority of NTDs could be prevented with FA supplementation before conception and in the first trimester of pregnancy [5]. Meanwhile, according to EUROCAT’s (the European network of population-based registries for the epidemiological surveillance of congenital anomalies) Special Reports, no progress in preventing NTDs in Europe was observed since then [7]; but there is still an on-going debate whether this remains the best strategy for increasing folate status. A serious disadvantage of this approach seems to be the lack of knowledge of proper daily diet supplementation with FA. As estimated, in Europe, half of pregnancies are unplanned. Meanwhile, NTDs occur in the first four weeks of pregnancy when most women do not know that they are pregnant. Even in planned pregnancies, many mothers do not take FA supplements to increase folate status before conception and for first twelve week of pregnancy [7,38], and the percentage of women who do, however, take dietary supplements at the right time, cannot be sure whether they obtain an authentic product with the amount of FA declared on the supplement label, as confirmed by the results of our study. The problem, therefore, appears to be more complex.

According to the Polish Society of Gynecologists and Obstetricians Guidelines, during supplementation, preparations with a documented composition and effect should be taken into account [17]. Consumers can purchase FA in the form of an over-the-counter drug in a dosage of, for example, 400 µg [9,10]. However, these are not as popular as commonly advertised supplements. Another strategy to increase the FA intake across the population is to introduce the obligation to fortify certain foods with the synthetic form of this vitamin: mandatory fortification of wheat flour has been implemented in the United States of America (USA) since 1998, and at present, more than 80 countries worldwide share this public health measure; nevertheless, none of them belong to the European Union (EU) [41]. Mandatory fortification has proved to be a better strategy for its widespread distribution across the population from children to adults, as long as wheat flour is consumed; but this measure has also been called a “double-edged sword strategy”, as excessive FA intakes could promote certain types of cancer amongst non-targeted population groups (e.g., the elderly) [42]. Conversely, a recent metanalysis by Moazzen et al. found no significant incidence of overall colorectal cancer risk in the population consuming FA [43].

FA, as the synthetic form of B9 folate vitamers, shows a higher bioavailability and, thus, a greater potential for improving folate status [44]. In a recent review by Garret and Bailey [45], the authors underlined that large-scale, mandatory FA fortification is an evidence-based intervention that effectively reduces the prevalence of NTDs, and that it is still underutilized in low- and middle-income countries and should be a main component of public health strategies targeted at NTD prevention.

While European countries remain reluctant to introduce widespread mandatory fortification of cereal flours such as wheat flour, the market of FA voluntary fortified foods is also developing towards offering, for instance, cereal-based products (e.g., ready-to-eat breakfast cereals) and fruit and vegetable juices with the addition of synthetic FA. However, recent studies on FA voluntary fortified products in Europe already indicated the problem of their authenticity. Results demonstrate that both too little or too much uncontrolled addition of the vitamin might be a common practice of manufacturers [46,47]. However, the most important, and at the same time safe and effective, strategy for all population groups—to increase natural folate intake—cannot be forgotten and undervalued [48]. The consumption of naturally occurring folate-rich food should be promoted and be a basic element of a combined strategy with supplemental folate; namely, the Mediterranean diet includes many food groups which are excellent sources of folates and of other health-promoting nutrients, with the potential to reduce chronic disease risk [48,49]. Data analysis of Sicińska and Wyka [29] showed that among the Polish population, the main sources of dietary folate were vegetables and bread, while using FA supplements was declared by 18–25% of young non-pregnant women aged 18–35, and merely 13–14% of the elderly.

## 4. Conclusions

There is a strong need for a combined strategy to promote folate intake increase in various population groups. Education on the proper intake of FA supplements, especially in women of childbearing age, and the promotion of foods naturally rich in folates, should be at the basis of this strategy. However, neither the strategy of using dietary supplements, nor increasing consumption of foods enriched with FA, will bring the expected results unless these products are authentic and comply with declared values.

The conducted studies on FA supplements confirmed the urgent need to introduce legislative changes which will allow greater control of the dietary supplements market by Sanitary Inspections. An effective control system to confirm the authenticity of supplements initially placed on the market and already available on sale, as well as an efficient withdrawal procedure of adulterated products are needed. Increasing notification fees and fines for placing products on the market in which actual composition is different than that declared on the label, can help protect the consumer from unfair practice.

## Figures and Tables

**Table 1:** Polish standards for the recommended daily allowance (RDA) and adequate intake (AI) for folates.

Gender and Age	Folate Equivalent (µg/day)	
	RDA	AI
Infants		
0–6 months		65
7–11 months		80
Children		
1–3	150	
4–6	200	
7–9	300	
Teenagers		
10–12	300	
13–18	400	
Adults		
19–75	400	
Pregnant women	600	
Lactating	500	

Based on Jarosz et al., 2017 [8].

**Table 2:** Indications for the use of dietary supplements containing folic acid provided by the manufacturers on product packaging.

Intended Use	No. of Products	Declared Folic Acid Content (µg per Dose)	
		Min-Max	Mode *
**For adults**			
Supplementation of the daily diet	164	16–1000	200
Proper heart function, maintenance of normal cholesterol levels and homocysteine metabolism	29	30–600	200
Promotes proper vision	6	100–400	200
Increased physical and mental effort	19	40–400	200
Supporting the functioning of the nervous system, supporting the work of the brain	11	200–400	200
**For women**			
For women (general)	21	100–400	200
For women planning and/or pregnant and breastfeeding	76	100–800	400
Better condition of skin, hair and nails	28	25–600	200
For women over 50 and going through menopause	12	20–400	400
**For men**	18	133–400	200
**For children**	36	12.5–300	100
**For the elderly**	14	100–400	100

* mode—the most common amount of folic acid (µg per dose).

**Table 3:** Folic acid (FA) content determined and declared in the tested dietary supplements.

Product No.	Determined FA Content	Declared FA Content (DV ^1^)	Difference with the Declared FA Content
	**One-ingredient supplements** **(µg per tablet, capsule)**		**[%]**
1	296.5^2^ ± 8.4	400	25.9
2	374.6 ± 9.3	400	6.4
3	374.5 ± 10.8	400	6.4
4	290.4 ± 22.7	400	27.4
5	292.5 ± 20.5	400	26.9
6	361.1 ± 2.9	400	9.7
7	320.2 ± 26.6	400	20.0
8	304.0 ± 14.7	400	24.0
	**Multi-ingredients supplements** **(µg per tablet, capsule)**		**[%]**
9	3.8 ± 0.3	400	99.1
10	339.2 ± 13.5	400	15.2
11	327.2 ± 20.4	400	18.2
12	3.6 ± 0.2	400	99.1
13	193.9 ± 9.1	600	67.7
14	314.7 ± 28.1	400	21.3
15	371.6 ± 48.4	600	38.1
16	209.9 ± 8.57	400	47.5
17	258.2 ± 1.79	400	35.5
18	250.4 ± 6.8	300	16.5
19	195.2 ± 8.94	200	2.4
20	167.3 ± 2.56	200	16.4
	**Multi-ingredients liquid supplements** **(µg per portion mL)**		**[%]**
21	204.1 ± 7.7	200	+2.0
22	10.7 ± 0.7	30	64.4
23	87.8 ± 3.4	180	51.2
24	173.4 ± 4.1	200	13.3
25	80.6 ± 3.1	100	19.4
26	65.1 ± 0.9	147	55.7
27	1.2 ± 0.1	50	97.5
28	184.1 ± 17.9	200	8.0
29	1.2 ± 0.1	169	99.3
30	57.9 ± 1.0	290	80.0

^1^ DV: Folic acid content declared by manufacturers or declared value. Results are presented as the mean of three replicates ± standard deviation.

## Data Availability

Data is contained within the article.
